# Investigating the Transcriptome of *Candida albicans* in a Dual-Species *Staphylococcus aureus* Biofilm Model

**DOI:** 10.3389/fcimb.2021.791523

**Published:** 2021-11-23

**Authors:** Bryn Short, Christopher Delaney, Emily McKloud, Jason L. Brown, Ryan Kean, Gary J. Litherland, Craig Williams, S. Lorraine Martin, William G. MacKay, Gordon Ramage

**Affiliations:** ^1^ Institute of Biomedical and Environmental Health Research, School of Health and Life Sciences, University of the West of Scotland, Paisley, United Kingdom; ^2^ School of Medicine, Dentistry and Nursing, University of Glasgow, Glasgow, United Kingdom; ^3^ Glasgow Biofilms Research Network, Glasgow, United Kingdom; ^4^ Department of Biological and Biomedical Sciences, School of Health and Life Sciences, Glasgow Caledonian University, Glasgow, United Kingdom; ^5^ Centre for Experimental Medicine, Queen’s University Belfast, Belfast, Ireland

**Keywords:** *Candida*, *Staphylococcus*, biofilm, transcriptomics, interkingdom interactions

## Abstract

*Candida albicans* is an opportunistic pathogen found throughout multiple body sites and is frequently co-isolated from infections of the respiratory tract and oral cavity with *Staphylococcus aureus.* Herein we present the first report of the effects that *S. aureus* elicits on the *C. albicans* transcriptome. Dual-species biofilms containing *S. aureus* and *C. albicans* mutants defective in *ALS3* or *ECE1* were optimised and characterised, followed by transcriptional profiling of *C. albicans* by RNA-sequencing (RNA-seq). Altered phenotypes in *C. albicans* mutants revealed specific interaction profiles between fungus and bacteria. The major adhesion and virulence proteins Als3 and Ece1, respectively, were found to have substantial effects on the *Candida* transcriptome in early and mature biofilms. Despite this, deletion of *ECE1* did not adversely affect biofilm formation or the ability of *S. aureus* to interact with *C. albicans* hyphae. Upregulated genes in dual-species biofilms corresponded to multiple gene ontology terms, including those attributed to virulence, biofilm formation and protein binding such as *ACE2* and multiple heat-shock protein genes. This shows that *S. aureus* pushes *C. albicans* towards a more virulent genotype, helping us to understand the driving forces behind the increased severity of *C. albicans*-*S. aureus* infections.

## Introduction

*Candida albicans* is typically found as a commensal organism at mucosal and barrier sites, such as the oral cavity, respiratory and gastrointestinal tracts. Under certain conditions, *C. albicans* is capable of causing opportunistic infections, ranging from superficial to systemic disease, making it one of the most common fungal infections (Brown et al., 2012). The mechanism behind the opportunistic nature of this pathogen lies in its ability to transition from a budding yeast cell to a highly invasive and filamentous hyphal cell, which are typically associated as being commensal and pathogenic phenotypes, respectively (Brown et al., 2012; Lu et al., 2014). Whether acting as a commensal or pathogen, *C. albicans* frequently co-exists with various bacterial species at mucosal sites, and the clinical significance of polymicrobial infections is becoming more apparent (Delaney et al., 2018). Interactions that take place within these infections can be synergistic, leading to traits such as increased drug resistance, virulence and biofilm formation (Harriott and Noverr, 2011; Kean et al., 2017).

*C. albicans* is frequently implicated in polymicrobial infections with bacteria such as *Staphylococcus aureus, Staphylococcus epidermidis* or *Pseudomonas aeruginosa* (Peters et al., 2012a; Haiko et al., 2019). A considerable amount of infections involving *C. albicans* and *S. aureus* are linked to biofilms in conditions such as angular chelitis, cystic fibrosis and diabetic foot ulcers (Peters et al., 2012a; Tsui et al., 2016).

The relationship between *C. albicans* and *S. aureus* appears to be beneficial for the bacterium, which utilises the fungi to augment its own virulence and resistance capabilities. *S. aureus* has been reported to coat itself in the *C. albicans* extracellular component, β-1,3-glucan in order to increase tolerance to vancomycin (Kong et al., 2016). Our group has also demonstrated that *C. albicans* increases the virulence of *S. aureus* in the *Galleria mellonella* infection model (Kean et al., 2017). Similar increases in virulence in animal infection models have also been shown (Peters and Noverr, 2013; Todd et al., 2019). Indeed, in a murine infection model, *C. albicans* was shown to have an ability to augment the *agr* quorum sensing system of *S. aureus*, resulting in increased alpha- and delta-toxin production, bacterial burden and mortality rates (Wang et al., 2007; Otto, 2014; Todd et al., 2019). Moreover, because the bacteria adhere directly to the fungal hyphae, *S. aureus* can more readily invade host cells through its close association with the invasive hyphae, which is akin to a needle-stick injection. In the context of complex multispecies communities, *C. albicans* has been described as a ‘keystone commensal’, suggesting it plays a critical physical role in promoting and maintaining biofilm stability in complex communities (Janus et al., 2016; Young et al., 2020).

When interacting with *C. albicans*, *S. aureus* preferentially adheres to the agglutinin like sequence 3 protein (Als3) (Peters et al., 2012b), which is highly expressed during early stages of *C. albicans* filamentous growth (Sherry et al., 2014). The Als3 protein plays multiple roles in the *C. albicans* infection cycle. These include the initial adhesion to host epithelial cells which subsequently induces its own endocytosis (Phan et al., 2007). As well as binding to host tissues, Als3 mediates self-adherence as well as *Candida-*bacteria binding as deletion of *ALS3* results in sparse and disorganised biofilms (Nobile et al., 2006a; Peters et al., 2012b). These *Candida-*bacteria interactions may be reciprocal, as it has been reported that *C. albicans* Als3 shares over 80% homology with *S. aureus* collagen binding factor (Sheppard et al., 2004). Several other genes including, but not limited to, hyphal wall protein 1 (*HWP1*), enhanced filamentous growth protein 1 (*EFG1*) and extent of cell elongation 1 (*ECE1*) have been described as being crucial to *C. albicans* pathogenesis, and appear to be co-expressed alongside *ALS3* (Ramage et al., 2002; Nobile et al., 2006b; Moyes et al., 2016). Of these genes, and arguably one of the most important, is *ECE1*, which encodes a 29 kDa cytolytic protein (named candidalysin) that is essential for virulence and epithelial cell damage in *C. albicans* infections. Cells lacking *ECE1* show no identifiable morphological differences, do not trigger epithelial cell danger response and are avirulent in animal infection models (Moyes et al., 2016). Recent work has revealed that the global repressor Tup1 and transcription factor Ahr1 are both required for expression of *ALS3* and *ECE1* (Ruben et al., 2020). Together these data highlight the importance of gene networks controlling pathogenicity, but we are still unclear on how these are controlled in dual-species interactions. Therefore, more in-depth analyses of these interactions are required.

Taken together, it is clear that there is a lack of knowledge surrounding the behaviour of *C. albicans* in the presence of *S. aureus.* Therefore, we aimed to address these gaps in the literature regarding the transcriptomic response of *C. albicans* to *S. aureus* in a dual-species biofilm using a combination of phenotypic and microscopic analyses in combination with RNA-seq. Secondly, we sought to determine the role that key virulence genes, *ALS3* and *ECE1* play in the regulation of these interactions.

## Materials and Methods

### Microbial Storage and Standardisation

*Candida albicans* SC5314, *C. albicans als3*Δ/Δ (Silverman et al., 2010)*, C. albicans ece1*Δ/Δ (Moyes et al., 2016) and *Staphylococcus aureus* NCTC 10833 were used in this study. *C. albicans* strains and *S. aureus* were grown on Sabourauds Dextrose (SAB) agar (ThermoFisher, Paisley, UK) and Luria Bertani (LB) agar (ThermoFisher), respectively and stored at 4°C. For long-term storage all organisms were stored in glycerol at -80°C.

To prepare overnight broths of each microorganism, one colony of *C. albicans* was suspended in Yeast Peptone Dextrose (YPD) media (ThermoFisher) and incubated at 30°C with agitation at 200 rpm. Luria Bertani broth (LB, ThermoFisher) was used to grow overnight broths of *S. aureus* at 37°C. Overnight cultures were washed by centrifugation and subsequent re-suspension in Phosphate Buffered Saline (PBS, Sigma-Aldrich, Dorset, UK). *S. aureus* cells were diluted to 0.6 OD_600_, equating to approximately 1x10^8^ cells/mL as determined by serial dilution and colony counting (data not shown). *C. albicans* concentrations were determined by cell counting on a Neubauer haemocytometer and diluted to 1x10^6^ cells/mL in growth media.

### Media Preparation

Todd Hewitt broth (THB, Sigma-Aldrich) was prepared and supplemented with 10 µM menadione and 4 mg/mL hemin (ThermoFisher) and mixed 1:1 with Roswell Park Memorial Institute media (RPMI). Referred to as sTHB from herein. A similar media has been described elsewhere and has been shown to enable the growth of both fungi and bacteria (Montelongo-Jauregui et al., 2016).

### Biofilm Growth and Analysis

Following counting of yeast cells and standardisation of bacteria, all cells were added to sTHB media to give a final concentration of 1x10^6^ CFU/mL. Biofilms were grown by adding inoculated growth media to the desired wells of a flat-bottomed, 96-well microtitre plate and *Candida* was incubated with or without the presence of bacteria for 4 or 24 h. Each type of biofilm was grown with 8 internal replicates and media only controls were included to test for contamination. Following the incubation step, biofilms were washed with PBS and then incubated with 0.05% crystal violet (CV) as described previously (Sherry et al., 2014). CV absorbance was measured at 570 nm using a multi-mode plate reader (FLUOStar Omega, BMG Labtech, Aylesbury, UK).

### DNA Extraction and Biofilm Composition Analysis

Early and mature biofilms of *C. albicans* mutants with and without *S. aureus* were grown on polymer coverslips before removing biomass *via* sonication at 35kHz in an ultrasonic water bath in 1 mL PBS for 10 minutes. DNA was extracted from biofilm cells using the Qiagen DNA mini-kit (Qiagen, Hilden, Germany) following the manufacturer’s instructions. Quantitative PCR (qPCR) was then used to determine the total number of cells within each biofilm as described by Kean et al. (2017). qPCR was carried out using the Step-One plus real time PCR machine (Life Technologies, Paisley, UK). The following profile was used: 50°C for 2 min, 95°C for 2 min, followed by 40 cycles of 95°C for 3 s and 60°C for 30 s. Colony forming equivalents (CFE) were calculated compared to a standard curve of serially diluted DNA of each species as previously described (O’Donnell et al., 2016). Species-specific primer sequences are provided in **Table 1**.

**Table 1:** Species specific primers used to identify *C. albicans* and *S. aureus*.

Target Organism	Forward Sequence (5’ – 3’)	Reverse Sequence (5’ – 3’)	Reference
*Candida albicans*	*GAGCGTCGTTTCTCCCTCAAACCGCTGG*	*GGTGGACGTTACCGCCGCAAGCAATGTT*	(Kean et al., 2017)
*Staphylococcus aureus*	*ATTTGGTCCCAGTGGTGTGGGTAT*	*GCTGTGACAATTGCCGTTTG TCGT*	(O’Donnell et al., 2016)

### Visualisation of Inter-Kingdom Biofilm Interactions

*C. albicans* was standardised to 1x10^6^ cells/mL, as described above, and biofilms grown in chamber slides (ThermoFisher) for 2 h at 37°C to induce hyphal formation. Bacteria were standardised to approximately 1x10^8^ cells/mL, stained with 1.5 mM hexidium iodide (ThermoFisher) and incubated at 37˚C for 1 h. Bacterial cells were pelleted by centrifugation and washed twice with PBS. *Candida* biofilms were washed with PBS following initial incubation. Bacterial cells at 5x10^7^ cells/mL and 1.5 mM calcofluor white (Sigma-Aldrich) were added to the chamber slide for a further hour at 37°C. After a total growth time of 3h, biofilms were then washed with PBS and imaged using an EVOS cell imaging system (ThermoFisher). Calcofluor white and hexidium iodide fluorescence was detected at excitation/emission wavelengths of 357/447 and 531/593 nm, respectively, before overlaying the images.

### RNA Extraction and Sequencing

Dual-species biofilms of *C. albicans* and *S. aureus* were grown for 4 and 24 h in 1:1 broth in T-75 cell culture flasks (Corning, UK). At each time point, media was removed and PBS was used to remove any non-adherent cells. Biofilm biomass was harvested using a cell scraper and stored in 1mL RNA later (ThermoFisher). Extractions were performed using RiboPure RNA Extraction Kits (ThermoFisher) following the manufacturer’s instructions. RNA quality and quantity was assessed using a Bioanalyser (Agilent, USA), where a minimum RNA integrity number (RIN) of 7.0 and a minimum quantity of 2.5 µg was achieved for each sample. RNA was sequenced by Edinburgh Genomics (genomics.ed.ac.uk) using a NovaSeq 6000 platform to provide 100bp paired end reads.

FastQC was used to assign quality scores to the produced reads and Illumina adaptors and poor-quality reads were trimmed using Trimmomatic. HISTAT2 was then used to align the resulting reads to a reference *C. albicans* genome (candidagenomedatabase.org) Assembly22 before the number of sequences that were aligned to each gene were counted using HTSeq. The counted genes were subsequently imported to RStudio (version 3.6.3) in which, the DESeq2 package was used to analyse the differentially expressed genes.

### Transcriptome Validation

Firstly, single and dual-species biofilms were grown on coverslips for 24h as described above. Following the growth phase, Biomass was removed by sonication and suspended in 1mL PBS. As per the manufacturers instructions, RNA was extracted using the RNeasy mini kit (Qiagen) and then converted into cDNA using High-Capacity cDNA Reverse Transcription Kit (ThermoFisher). qPCR was carried out using the Step-One plus real time PCR machine (Life Technologies). The following profile was used: 50°C for 2 min, 95°C for 2 min, followed by 40 cycles of 95°C for 3 s and 60°C for 30 s. Expression of each gene-of-interest was measured in relation to expression of *ACT1* before comparisons between single and dual-species biofilms. Gene primer sequences are provided in **Supplementary Table 1**.

### Statistical Analysis

Figures depicting differential gene expression between *C. albicans* and *S. aureus* biofilms were created using the DESeq2 package in RStudio. Data was also visualised as heatmap as and volcano plots utilising R packages pheatmap and EnhancedVolcano. Principle component analysis (PCA) was performed within R to visualise the dimension within the gene expression data which correspond to the most variance.

Gene interaction networks were created using the ClueGO application in Cytoscape (cytoscape.com). All other graphs and analyses were performed in GraphPad Prism (version 7, La Jolla, California, USA). Non-parametric Kruskal-Wallis tests were used to compare means of corrected raw data following biofilm assays followed by Dunn’s test for multiple comparisons. Differences between means were deemed significant where *P* < 0.05 and a minimum Log2 fold-change of ±1.5 was applied when analysing gene expression data.

## Results

**Figure 1:**
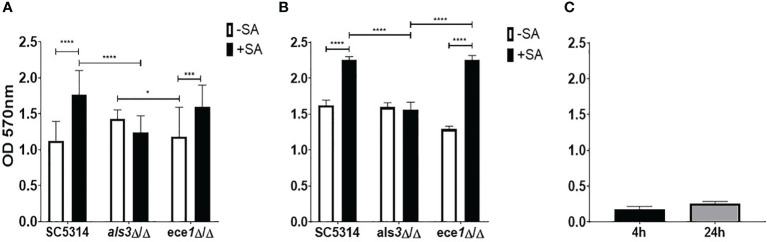
Als3 is responsible for *Candida-Staphylococcus* interactions. Single and multi-species biofilms containing *C. albicans* and *S. aureus* were grown for **(A)** 4 h and **(B)** 24 h; **(C)** single species *S. aureus* biofilm only. Biomass was quantified by staining using 0.05% crystal violet. Experiments were performed on three separate occasions and error bars represent standard deviation of the mean (*; *P* < 0.05, ***; *P* < 0.001, ****; *P* < 0.0001).

In addition to *S. aureus*, other staphylococcal species such as *Staphylococcus epidermidis* has displayed an ability to interact synergistically with *C. albicans* (El-Azizi et al., 2004; Pammi et al., 2013). The strain of *S. aureus* used herein was selected purposefully and it lacks the full repertoire of biofilm-forming genes and binds preferentially to *C. albicans* hyphae, increasing confidence that observed changes in *C. albicans* is a result of direct fungal-bacterial interactions. *ALS3* has been shown to significantly influence staphylococcal interactions and expression of this gene has been linked to the expression of the key virulence gene, *ECE1* (Peters et al., 2012b; Ruben et al., 2020). Therefore, to begin identifying the role these genes play in the interkingdom interactions we investigated the impact that losing one of these key biofilm genes has on dual-species biofilm formation with *S. aureus.* Biofilms were grown for 4 and 24 h as mono- or dual species biofilms with *S. aureus* and crystal violet (CV) was used to assess total biomass (**Figure 1**). The presence of *S. aureus* resulted in significant increases (*P* < 0.0001) in dual-species biofilm biomass when grown with both WT and *ece1*Δ/Δ *C. albicans* at both 4 and 24 h (**Figures 1A, B**). No significant change in biomass was observed in *als3*Δ/Δ or *S. aureus* only (**Figures 1A–C**) biofilms regardless, of biofilm maturity. No considerable differences in fungal cell morphology were observed, regardless of genotype (data not shown). As expected, *S. aureus* produced a poor biofilm following 4 and 24 h growth (**Figure 1C**).

**Figure 2:**
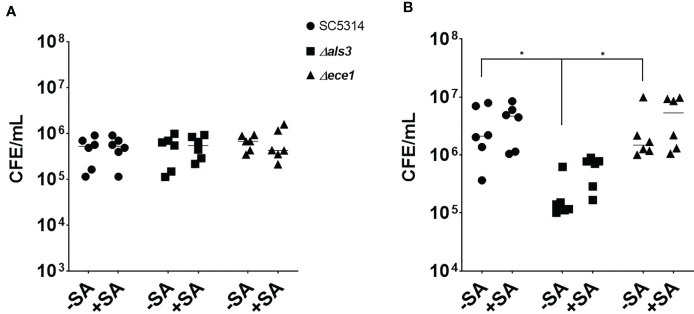
*Staphylococcus aureus* does not influence the total number of fungal cells in a dual-species biofilm. Biofilm biomass was removed *via* sonication, DNA was extracted and the total number of fungal cells in each biofilm was quantified using qPCR. The total number of *C. albicans* cells of **(A)** 4 h and **(B)** 24 h biofilms are presented as colony forming equivalents per mL (CFE/mL). Experiments were repeated three times on three separate occasions. Data points represent individual biofilms. CFEs of *C. albicans* SC5314 and *ece1*Δ/Δ dual-species biofilms were compared to that of *als3*Δ/Δ (*, *P* < 0.05).

Given the changes associated with biofilm biomass, the quantity of each organism in each biofilm was assessed using qPCR (**Figure 2**). The number of *C. albicans* cells in 4 h biofilms remained approximately 5x10^5^ cells/mL regardless of presence or absence of *S. aureus* (**Figure 2A**). The average number of WT and *ece1*Δ/Δ *C. albicans* in 24 h biofilms increased from 4.8x10^5^ to 3.4x10^6^ and 6.5x10^5^ to 2.8x10^6^ cells/mL (equivalent to a 6.9 and 4.2 fold increase), respectively. As expected, there were significantly fewer *C. albicans als3*Δ/Δ cells compared to the other *C. albicans* strains (*P* < 0.05; **Figure 2B**). Although there were differences in the total number of *C. albicans* cells when comparing one strain to another, the presence of *S. aureus* did not affect the number of fungal cells in any biofilm. The significant increases in biomass thus suggested that there must be an increase in *S. aureus* colonising the biofilm, which was confirmed by the quantification of total bacterial cells in the dual-species biofilms (**Supplementary Figure S1**). In early dual-species biofilms, the number of *S. aureus* cells in biofilms with *C. albicans als3*Δ/Δ was 100 and 345-fold lower than the WT and *ece1*Δ/Δ strains, respectively (*P* < 0.05; **Supplementary Figure S1A**). A similar trend was observed at 24 h, where the concentration of *S. aureus* recovered from biofilms grown with WT and the *ece1*Δ/Δ strains was significantly higher than that of *als3*Δ/Δ (*P* < 0.05).

**Figure 3:**
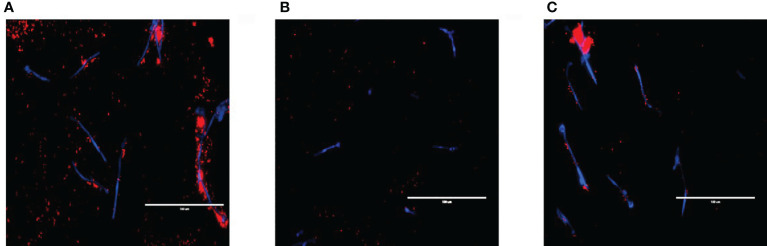
Visualising cell-cell interactions between *Candida* mutants and *S. aureus. C albicans* biofilms of **(A)** wild type SC5314 and **(B)** *ALS3* and **(C)** *ECE1* deletion mutants were grown for 2 h before adding 5x10^7^ cells/mL of *S. aureus* (which had been pre-stained with hexidium iodide for 1 h prior) for a further hour. Calcofluor white was added to the bacterial inoculum to stain the fungal biofilm before washing the biofilm to remove any non-adherent cells before imaging. Scale bars represent 100μm.

The relationship between *S. aureus* and each *C. albicans* strain was visualised using fluorescent microscopy. It was observed that *S. aureus* was able to avidly bind to *C. albicans* hyphae of WT and *ece1*Δ/Δ strains (**Figure 3**) (additional images are provided as part of supplementary data; **Supplementary Figure S2**). As expected, deletion of *ALS3* considerably influenced the relationship between *C. albicans* and *S. aureus*, resulting in a decrease in the ability of *S. aureus* to integrate into the *C. albicans* biofilm. *S. aureus* integration was comparable between WT and *ece1*Δ/Δ strains. Taken together, **Figures 1**–**3** show that when forming dual-species biofilms, *S. aureus* is closely associated with *C. albicans* hyphae which is mediated by Als3.

**Figure 4:**
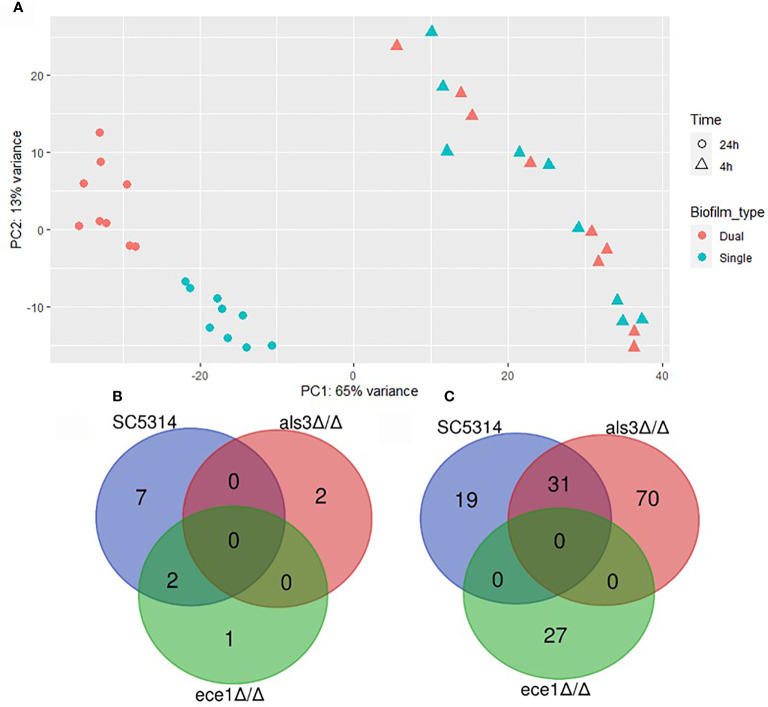
Presence of *Staphylococcus aureus* determines mature biofilm transcriptome. **(A)** Principal component analysis plot shows distinct grouping of 24 h samples with the variable of largest variance along PC1 (65%) and second largest along PC2 (13%). Venn diagrams show the number of genes upregulated in 24 h **(B)** single (*C. albicans* only) and **(C)** dual-species (*C. albicans* and *S. aureus)* biofilms.

Differential expression (DE) analysis was performed to identify transcriptional changes in *C. albicans* biofilms when interacting with *S. aureus.* Multivariate analysis by principal component analysis (PCA) showed variance between samples by biofilm maturity and presence of *S. aureus* (**Figure 4A**). The greatest amount of variance is observed between 4 h and 24 h with a large number of differentially expressed genes being identified (**Supplementary Figure S3**). Limited numbers of genes with increased expression were observed in single species biofilms with only 12 upregulated genes in total being specific to *C. albicans* only biofilms (**Figure 4B**). Each dual-species biofilm presented its own distinct patterns of up-regulation with 50 and 101 upregulated genes in WT and *als3*Δ/Δ dual species biofilms (**Figure 4C**). A depleted response of the *ece1*Δ/Δ strain was observed with only 27 upregulated genes identified and only 5 of these were specific to the strain. From these data, it can be concluded that any significant, strain specific changes in *C. albicans* transcription occurred within mature biofilms, and therefore, future analyses were limited to the 24 h time point. Full lists of upregulated genes in single and dual-species biofilms are provided in **Supplementary Tables 2**–**5**.

**Figure 5:**
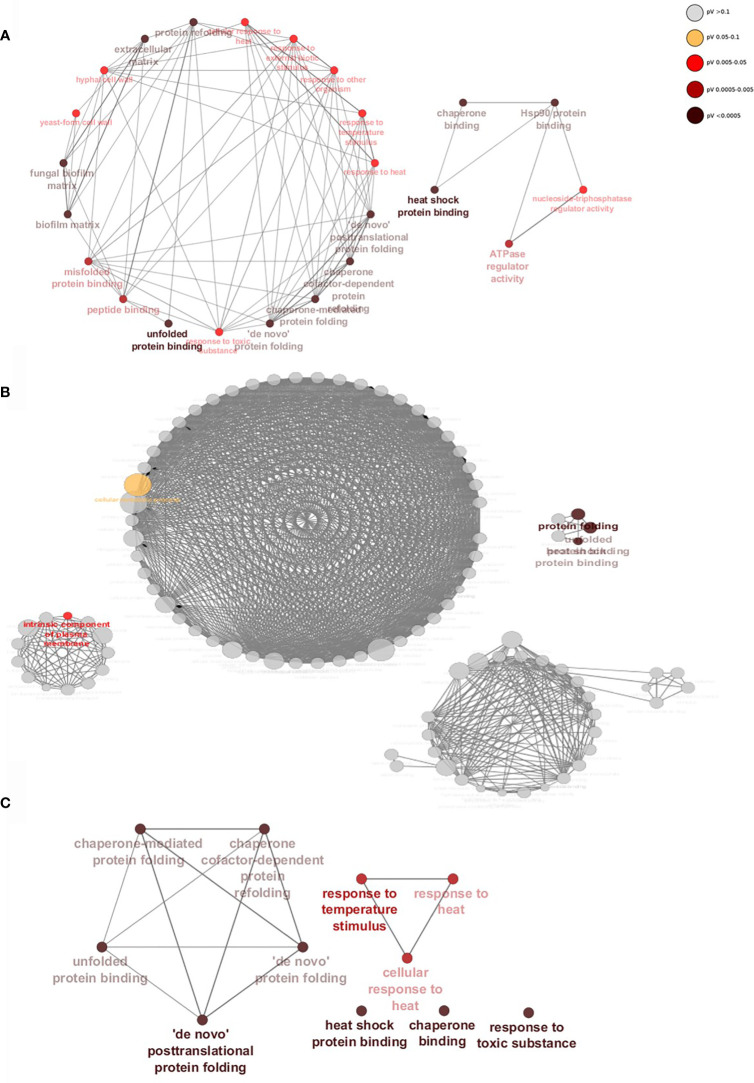
*Staphylococcus aureus* induces significant upregulation of biofilm-associated genes when binding to Als3. Gene networks show interactions between upregulated genes specific to **(A)** WT, **(B)** *ALS3* and **(C)** *ECE1* null mutant dual-species, mature biofilms. Genes of similar function are grouped together to form nodes (circles) and nodes with similar functions are linked by edges (lines). Nodes are coloured by levels of significance and node size increases with the number of genes involved in each function. Networks were created using ClueGO.

Gene interaction networks show that within a 24h dual-species biofilm there is an upregulation of genes of related function (**Figure 5**). Genes are grouped by function, taking into account their gene ontology (GO), which forms the nodes (circles) and nodes with similar or related functions are joined by edges (lines). When binding to WT *C. albicans* (**Figure 5A**), *S. aureus* stimulates expression of several genes divided into two distinct groups. Genes in the larger network are classified by functions such as fungal cell wall, biofilm matrix and peptide binding (*P* < 0.05). As described above, deletion of *ALS3* had noticeable effects on biofilm formation, resulting in a vastly different gene expression profile and differentially expressed genes in these biofilms were divided into 4 unrelated groups (**Figure 5B**). Despite there being a high number of upregulated genes specific to the dual-species *als3*Δ/Δ biofilm, only 5 gene nodes reached statistical significance (*P* < 0.05). Genes composing these significant nodes are implicated in processes such as cellular metabolism, protein folding and plasma membrane components. A highly significant yet limited response in genes related to stress responses and protein binding and folding was observed in *C. albicans ece1*Δ/Δ cells (**Figure 5C**).

**Figure 6:**
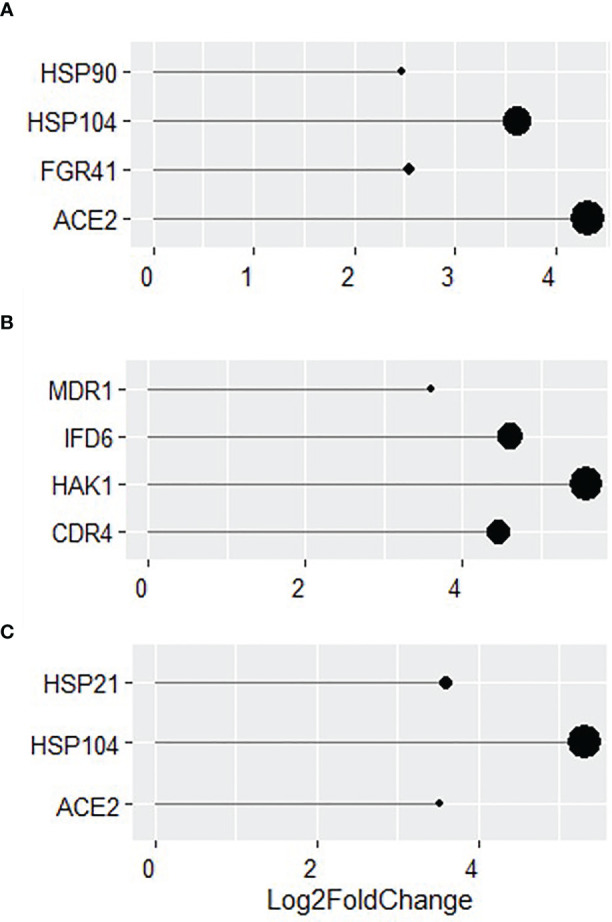
Identification of key genes with increased expression in dual-species biofilms. Log_2_ fold change of key genes in 24 h dual-species biofilms of **(A)** WT, **(B)** *als3*Δ/Δ and **(C)** *ece1*Δ/Δ *C albicans* with *S. aureus*.

More in-depth analyses of transcriptional changes at individual gene levels revealed a number of genes involved in biofilm formation and virulence (*HSP90, HSP104, FGR41 and ACE2;* **Figure 6A**) to be highly upregulated in WT *C. albicans* (all over a Log_2_ fold change of 2). The increased expression of genes related to the *als3*Δ/Δ strain were found to be typically upregulated in response to external stressors. Some of the genes with the highest increased expression were *MDR1, IFD6, HAK1* and *CDR4* with a Log_2_ fold increase of 3.6, 4.6, 5.5 and 4.4, respectively (**Figure 6**). Genes involved in virulence and biofilm formation (*HSP21, HSP104* and *ACE2*) were also upregulated by more than a Log_2_ fold increase of 3 in *ece1*Δ/Δ dual-species biofilms, much like in the WT biofilm (**Figure 6C**). These findings were confirmed by qPCR analysis (**Supplementary Figure S4**). **Figures 5**, **6** show that the response of *C. albicans* to *S. aureus* is considerably different when the bacterium is unable to bind to Als3, resulting in the upregulation of stress response genes. **Figures 5**, **6** suggest that loss of Ece1 does not alter the surface interactions between these two nosocomial pathogens and although a limited transcriptional response is observed in the *ece1*Δ/Δ strains, 63% of upregulated genes are shared with the WT. Therefore, when bound to the preferred receptor of Als3, *S. aureus* augments *C. albicans* virulence through upregulation of virulence and biofilm associated genes.

## Discussion

The presence of polymicrobial biofilms during infection affects patient outcome due to decreased susceptibility to antimicrobial treatments and increased duration of hospital stay (Sancho et al., 2012). With increased understanding of the importance of interactions between fungi and bacteria, polymicrobial interkingdom biofilms have been identified in increasing numbers during infection. Of these interkingdom biofilms, *C. albicans* and *S. aureus* are frequently co-isolated from conditions such as cystic fibrosis and periodontitis (Valenza et al., 2008; Carolus et al., 2019). We now have a better understanding of the mechanisms by which these interactions benefit *S. aureus* (Harriott and Noverr, 2009; Kong et al., 2016; Todd et al., 2019) but what remains unclear is how this relationship affects *C. albicans*. The work carried out herein has therefore aimed to define the *C. albicans* transcriptome whilst interacting with *S. aureus.*

Our findings from analysis of dual-species biofilms describe a synergistic relationship between both organisms. Similar findings have been shown by our group in a previous study (Kean et al., 2017), whereby we showed that *C. albicans* augments bacterial growth through acting as a biofilm substrate to enhance bacterial colonisation, a process we defined as a mycofilm. These findings were confirmed here through biofilm compositional analysis, which revealed a significant increase in bacterial CFEs whilst concentrations of fungal cells remained unchanged. Close interactions between *C. albicans* and *S. aureus* were observed in **Figure 3**. Loss of Ece1 did not appear to affect this relationship which is in line with that reported by Peters et al. (2012b) who identified Als3 as the main binding receptor for *S. aureus* and reported a significant decrease in the ability of *S. aureus* to bind to *C. albicans* hyphae following *ALS3* deletion. These findings were more recently confirmed by (Van Dyck et al., 2021).

Although lacking the ability to elicit a proper immune response in the host, *ece1*Δ/Δ is still capable of forming robust biofilms (as observed in **Figure 1**) due to the significant up regulation of protein folding and binding genes as shown by the gene interaction networks in **Figure 5**. The same strain was also found to not exhibit any significant morphological differences to WT *C. albicans* (Moyes et al., 2016). It could be hypothesised that these cells may also prove to be more tolerant to external stimuli such as heat, which can be inferred from the significant upregulation of multiple heat-shock genes (*HSP104, HSP21* and *HSP70*).

Data presented in **Figure 4** showed that a limited number of transcriptional changes occurred in *ece1*Δ/Δ cells in comparison to the WT. Based on previous data describing Ece1 as a key virulence protein, it was hypothesised that transcriptional changes would mirror that of WT *C. albicans*. However, there were considerably fewer differentially regulated genes in the *ECE1* null mutant than other strains. Transcriptional analysis revealed that *PGA4* was downregulated in single-species *ece1*Δ/Δ biofilms when compared to WT (**Figure S5**). Expression of *PGA4* is required for proper cell wall biosynthesis whilst playing a minor role in regulating responses to antibiotics (Ene et al., 2012). As shown in **Figure S6**, expression of *PGA4* is linked to expression of several other cell wall related genes such as *CHT2, ECM331, BGL2* and *MP65* (Heilmann et al., 2011; Gil-Bona et al., 2018). This differential cell wall makeup may provide insight as to why the transcriptome of *ECE1* null strains responds differently to *S. aureus* compared to WT *C. albicans.*

Binding of *S. aureus* to WT and *ece1*Δ/Δ *C. albicans* induces upregulation of several genes whose functions are closely related to biofilm and hyphal formation. Genes involved in these processes include virulence, adhesion and filamentation genes such as *HSP90. HSP90* is the most commonly studied heat shock protein in *C. albicans*, due to its involvement as a transcription factor in several virulence associated pathways such as biofilm formation and hyphal morphogenesis (O’Meara et al., 2017). Other key upregulated genes include *HSP104, FGR41* and *ACE2*, which also play key roles in effective formation of hyphae and biofilms (Kelly et al., 2004; Fiori et al., 2012; Lan et al., 2017).

GO term analysis of WT strains revealed multiple groups of genes whose functions are related to biofilm formation such as peptide binding and extra-cellular matrix (ECM) formation. ECM is a key factor behind the recalcitrant nature of biofilms towards antimicrobial therapy (Singh et al., 2017). As described by Kong et al. (2016), *C. albicans* ECM components protect *S. aureus.* It can therefore, be deduced from this that *S. aureus* induces an upregulation of genes involved in *C. albicans* biofilm and ECM production such as *GLX3* (Cabello et al., 2018) to protect itself and the fungal cells from antimicrobials. Recent work has also shown that not only does *S. aureus* promote ECM production in *C. albicans* but the fungi also decreases expression of *S. aureus Nuc. Nuc* cleaves extracellular DNA and promotes biofilm dispersal (Vila et al., 2021). From this it can be deduced that *C. albicans* promotes *S. aureus* ECM production in *S. aureus* and vice versa. This supports data presented in **Figure 1** and previous findings by Kean et al. (2017) who reported an increase in biofilm formation and virulence in a dual-species inoculum.

As mentioned above, despite deletion of *ALS3* significantly altering the binding pattern of *S. aureus* it was not abolished. This resulted in increased expression of over 100 highly interconnected genes involved in biological processes, such as response to drugs and cellular response to stress as demonstrated in **Figure 5**. Although several genes were upregulated in both WT and *als3*Δ/Δ dual-species biofilms, there was minimal crossover of enriched GO terms in gene networks. This suggests that even though similar genes are upregulated in each strain, deletion of *ALS3* significantly alters how *C. albicans* interacts with and responds to *S. aureus*. Among the genes up regulated were *CDR4, MDR1* and *CAP1.* Although there is no confirmed role for *CDR4*, it is upregulated in the *C. albicans* core stress response (Enjalbert et al., 2006). Similar to *CDR1, CDR4* belongs to the ABC superfamily of efflux pumps, which can lead to speculation that the protein encoded by *CDR4* is also involved in drug resistance. *MDR1* and *CAP1* are also induced by external stressors such as antifungal drugs and oxidative stress, respectively (Feng et al., 2018). Therefore, it can be deduced that when *S. aureus* binds to other adhesion proteins other than Als3, it triggers *C. albicans* stress response pathways. Although this stress does not appear to hinder hyphal formation, virulence may be attenuated through loss of Als3.

Despite this study accurately describing the effects of *S. aureus* on the transcriptome of *C. albicans*, it is not without its limitations. For example, this study used a biofilm-deficient strain of *S. aureus* to focus more on direct fungal-bacterial interactions. The use of a biofilm positive bacterial strain is likely to interact with the *C. albicans* strains used herein differently, therefore resulting in a different fungal response. This would then help create a more detailed picture of how *S. aureus* influences the *C. albicans* transcriptome. Additionally, transcriptomics is not without its drawbacks such as this study only captured the *C. albicans* transcriptome at 4 and 24 h, which may be significantly different to earlier or late time-points. However, the inclusion of additional permutations in transcriptomics experiments can significantly increase costs. Previous work by Cue and colleagues identified that the *S. aureus* strain herein presented a biofilm deficient phenotype *via* secretion of a heat-stable peptide (Cue et al., 2015). This peptide was shown to inhibit biofilm formation in other *S. aureus* strains and could therefore be a strain specific and contributing factor towards the transcriptomic response observed above.

To conclude, this study is the first to report on the changes in the *C. albicans* transcriptome caused by the closely associated bacterial pathogen, *S. aureus.* We describe, under normal circumstances in WT *C. albicans*, an upregulation of biofilm and virulence associated genes when *S. aureus* adheres to Als3 that likely leads to a more virulent phenotype. We also show that *ECE1* is not required for staphylococci to interact with *C. albicans* and that the relationship between these two organisms is beneficial for the bacterium as well as the yeast through upregulation of adhesion and biofilm formation genes. *S. aureus* induces the upregulation of virulence-associated genes in *ece1*Δ/Δ dual-species that may compensate for the loss of virulence that comes with the loss of candidalysin. However, further experiments are required to discern if a virulent phenotype is indeed restored. Exploring these interactions in more depth will continue to unveil additional mechanisms of interaction, which may help to explain their frequent co-isolation in biofilm-related infections and identify potential, novel antimicrobial therapies to combat these complex biofilm communities in a diverse range of clinically important contexts.

## Data Availability Statement

The datasets presented in this study can be found in NCBI-SRA under BioProject number PRJNA731052.

## Author Contributions

BS, CD, and EM participated in study design, experimental procedures and data analysis and were also responsible for preparation of the manuscript. JB, RK, GL, CW, and WM participated in study design and preparation of the manuscript. LM participated in preparation and critical appraisal of the manuscript. GR conceived the study, participated in study design and data analysis and was responsible for producing the final manuscript. All authors contributed to the article and approved the submitted version.

## Funding

BS is supported by a matched Ph.D. studentship provided by the University of the West of Scotland to support the Borders and Regions Airways Training Hub project (BREATH; INT-VA/045) which is funded by the European Union (EU), under the INTERREG VA Programme, managed by the Special EU Programmes Body. We would also like to acknowledge the funding support of the BBSRC Industrial CASE PhD studentship for Christopher Delaney (BB/P504567/1).

## Conflict of Interest

The authors declare that the research was conducted in the absence of any commercial or financial relationships that could be construed as a potential conflict of interest.

## Publisher’s Note

All claims expressed in this article are solely those of the authors and do not necessarily represent those of their affiliated organizations, or those of the publisher, the editors and the reviewers. Any product that may be evaluated in this article, or claim that may be made by its manufacturer, is not guaranteed or endorsed by the publisher.

## References

[B1] Brown G. D. Denning D. W. Gow N. A. R. Levitz S. M. Netea M. G. White T. C. (2012). Hidden Killers: Human Fungal Infections. Sci. Trans. Med. 4, 165rv13–165rv13. doi: 10.1126/science.1222236 23253612

[B2] Cabello L. Gómez-Herreros E. Fernández-Pereira J. Maicas S. Martínez-Esparza M. C. De Groot P. W. J.. (2018). Deletion of GLX3 in Candida Albicans Affects Temperature Tolerance, Biofilm Formation and Virulence. FEMS Yeast Res. 19. doi: 10.1093/femsyr/foy124 30476034

[B3] Carolus H. Van Dyck K. Van Dijck P. (2019). Candida Albicans and Staphylococcus Species: A Threatening Twosome. Front. Microbiol. 10. doi: 10.3389/fmicb.2019.02162 PMC6759544 31620113

[B4] Cue D. Junecko J. M. Lei M. G. Blevins J. S. Smeltzer M. S. Lee C. Y. (2015). SaeRS-Dependent Inhibition of Biofilm Formation in Staphylococcus Aureus Newman. PloS One 10, e0123027–e0123027. doi: 10.1371/journal.pone.0123027 25853849 PMC4390220

[B5] Delaney C. Kean R. Short B. Tumelty M. Mclean W. Nile C. J.. (2018). Fungi at the Scene of the Crime: Innocent Bystanders or Accomplices in Oral Infections? Curr. Clin. Microbiol. Rep. 5, 190–200. doi: 10.1007/s40588-018-0100-3

[B6] El-Azizi M. A. Starks S. E. Khardori N. (2004). Interactions of Candida Albicans With Other Candida Spp. And Bacteria in the Biofilms. J. Appl. Microbiol. 96, 1067–1073. doi: 10.1111/j.1365-2672.2004.02213.x 15078523

[B7] Ene I. V. Heilmann C. J. Sorgo A. G. Walker L. A. De Koster C. G. Munro C. A.. (2012). Carbon Source-Induced Reprogramming of the Cell Wall Proteome and Secretome Modulates the Adherence and Drug Resistance of the Fungal Pathogen Candida Albicans. Proteomics 12, 3164–3179. doi: 10.1002/pmic.201200228 22997008 PMC3569869

[B8] Enjalbert B. Smith D. A. Cornell M. J. Alam I. Nicholls S. Brown A. J. P.. (2006). Role of the Hog1 Stress-Activated Protein Kinase in the Global Transcriptional Response to Stress in the Fungal Pathogen Candida Albicans. Mol. Biol. Cell 17, 1018–1032. doi: 10.1091/mbc.e05-06-0501 16339080 PMC1356608

[B9] Feng W. Yang J. Yang L. Li Q. Zhu X. Xi Z.. (2018). Research of Mrr1, Cap1 and MDR1 in Candida Albicans Resistant to Azole Medications. Exp. Ther. Med. 15, 1217–1224. doi: 10.3892/etm.2017.5518 29434708 PMC5774345

[B10] Fiori A. Kucharíková S. Govaert G. Cammue B. P. A. Thevissen K. Van Dijck P. (2012). The Heat-Induced Molecular Disaggregase Hsp104 of Candida Albicans Plays a Role in Biofilm Formation and Pathogenicity in a Worm Infection Model. Eukaryotic Cell 11, 1012–1020. doi: 10.1128/EC.00147-12 22635920 PMC3416063

[B11] Gil-Bona A. Amador-García A. Gil C. Monteoliva L. (2018). The External Face of Candida Albicans: A Proteomic View of the Cell Surface and the Extracellular Environment. J. Proteomics 180, 70–79. doi: 10.1016/j.jprot.2017.12.002 29223801

[B12] Haiko J. Saeedi B. Bagger G. Karpati F. Özenci V. (2019). Coexistence of Candida Species and Bacteria in Patients With Cystic Fibrosis. Eur. J. Clin. Microbiol. Infect. Dis. Off. Publ. Eur. Soc. Clin. Microbiol. 38, 1071–1077. doi: 10.1007/s10096-019-03493-3 PMC6520323 30739228

[B13] Harriott M. M. Noverr M. C. (2009). Candida Albicans and Staphylococcus Aureus Form Polymicrobial Biofilms: Effects on Antimicrobial Resistance. Antimicrob Agents chemother 53, 3914–3922. doi: 10.1128/AAC.00657-09 19564370 PMC2737866

[B14] Harriott M. M. Noverr M. C. (2011). Importance of Candida-Bacterial Polymicrobial Biofilms in Disease. Trends Microbiol. 19, 557–563. doi: 10.1016/j.tim.2011.07.004 21855346 PMC3205277

[B15] Heilmann C. J. Sorgo A. G. Siliakus A. R. Dekker H. L. Brul S. De Koster C. G.. (2011). Hyphal Induction in the Human Fungal Pathogen Candida Albicans Reveals a Characteristic Wall Protein Profile. Microbiol. (Reading) 157, 2297–2307. doi: 10.1099/mic.0.049395-0 21602216

[B16] Janus M. M. Willems H. M. Krom B. P. (2016). Candida Albicans in Multispecies Oral Communities; A Keystone Commensal? Adv. Exp. Med. Biol. 931, 13–20. doi: 10.1007/5584_2016_5 27271681

[B17] Kean R. Rajendran R. Haggarty J. Townsend E. M. Short B. Burgess K. E.. (2017). Candida Albicans Mycofilms Support Staphylococcus Aureus Colonization and Enhances Miconazole Resistance in Dual-Species Interactions. Front. Microbiol. 8. doi: 10.3389/fmicb.2017.00258 PMC5322193 28280487

[B18] Kelly M. T. Maccallum D. M. Clancy S. D. Odds F. C. Brown A. J. P. Butler G. (2004). The Candida Albicans CaACE2 Gene Affects Morphogenesis, Adherence and Virulence. Mol. Microbiol. 53, 969–983. doi: 10.1111/j.1365-2958.2004.04185.x 15255906

[B19] Kong E. F. Tsui C. Kucharíková S. Andes D. Van Dijck P. Jabra-Rizk M. A. (2016). Commensal Protection of Staphylococcus Aureus Against Antimicrobials by Candida Albicans Biofilm Matrix. mBio 7, e01365–e01316. doi: 10.1128/mBio.01365-16 27729510 PMC5061872

[B20] Lan Y.-B. Huang Y.-Z. Qu F. Li J.-Q. Ma L.-J. Yan J.. (2017). Time Course of Global Gene Expression Alterations in Candida Albicans During Infection of HeLa Cells. Bosnian J. Basic Med. Sci. 17, 120–131. doi: 10.17305/bjbms.2017.1667 PMC5474105 28397609

[B21] Lu Y. Su C. Liu H. (2014). Candida Albicans Hyphal Initiation and Elongation. Trends Microbiol. 22, 707–714. doi: 10.1016/j.tim.2014.09.001 25262420 PMC4256103

[B22] Montelongo-Jauregui D. Srinivasan A. Ramasubramanian A. K. Lopez-Ribot J. L. (2016). An In Vitro Model for Oral Mixed Biofilms of Candida Albicans and Streptococcus Gordonii in Synthetic Saliva. Front. Microbiol. 7, 686–686. doi: 10.3389/fmicb.2016.00686 27242712 PMC4864667

[B23] Moyes D. L. Wilson D. Richardson J. P. Mogavero S. Tang S. X. Wernecke J.. (2016). Candidalysin is a Fungal Peptide Toxin Critical for Mucosal Infection. Nature 532, 64–68. doi: 10.1038/nature17625 27027296 PMC4851236

[B24] Nobile C. J. Andes D. R. Nett J. E. Smith F. J. Yue F. Phan Q. T.. (2006a). Critical Role of Bcr1-Dependent Adhesins in C. Albicans Biofilm Formation *In Vitro* and *In Vivo* . PloS Pathog. 2, e63. doi: 10.1371/journal.ppat.0020063 16839200 PMC1487173

[B25] Nobile C. J. Nett J. E. Andes D. R. Mitchell A. P. (2006b). Function of Candida Albicans Adhesin Hwp1 in Biofilm Formation. Eukaryotic Cell 5, 1604–1610. doi: 10.1128/EC.00194-06 17030992 PMC1595337

[B26] O'donnell L. E. Smith K. Williams C. Nile C. J. Lappin D. F. Bradshaw D.. (2016). Dentures are a Reservoir for Respiratory Pathogens. J. Prosthodontics 25, 99–104. doi: 10.1111/jopr.12342 26260391

[B27] O'meara T. R. Robbins N. Cowen L. E. (2017). The Hsp90 Chaperone Network Modulates Candida Virulence Traits. Trends Microbiol. 25, 809–819. doi: 10.1016/j.tim.2017.05.003 28549824 PMC5610082

[B28] Otto M. (2014). Staphylococcus Aureus Toxins. Curr. Opin. Microbiol. 17, 32–37. doi: 10.1016/j.mib.2013.11.004 24581690 PMC3942668

[B29] Pammi M. Liang R. Hicks J. Mistretta T.-A. Versalovic J. (2013). Biofilm Extracellular DNA Enhances Mixed Species Biofilms of Staphylococcus Epidermidis and Candida Albicans. BMC Microbiol. 13, 1–13. doi: 10.1186/1471-2180-13-257 24228850 PMC3833181

[B30] Peters B. M. Jabra-Rizk M. A. O'may G. A. Costerton J. W. Shirtliff M. E. (2012a). Polymicrobial Interactions: Impact on Pathogenesis and Human Disease. Clin. Microbiol. Rev. 25, 193–213. doi: 10.1128/CMR.00013-11 22232376 PMC3255964

[B31] Peters B. M. Noverr M. C. (2013). Candida Albicans-Staphylococcus Aureus Polymicrobial Peritonitis Modulates Host Innate Immunity. Infect Immun. 81, 2178–2189. doi: 10.1128/IAI.00265-13 23545303 PMC3676024

[B32] Peters B. M. Ovchinnikova E. S. Krom B. P. Schlecht L. M. Zhou H. Hoyer L. L.. (2012b). Staphylococcus Aureus Adherence to Candida Albicans Hyphae is Mediated by the Hyphal Adhesin Als3p. Microbiology 158, 2975. doi: 10.1099/mic.0.062109-0 22918893 PMC4083660

[B33] Phan Q. T. Myers C. L. Fu Y. Sheppard D. C. Yeaman M. R. Welch W. H.. (2007). Als3 Is a Candida Albicans Invasin That Binds to Cadherins and Induces Endocytosis by Host Cells. PloS Biol. 5, e64. doi: 10.1371/journal.pbio.0050064 17311474 PMC1802757

[B34] Ramage G. Vandewalle K. López-Ribot J. L. Wickes B. L. (2002). The Filamentation Pathway Controlled by the Efg1 Regulator Protein is Required for Normal Biofilm Formation and Development in Candida Albicans. FEMS Microbiol. Lett. 214, 95–100. doi: 10.1111/j.1574-6968.2002.tb11330.x 12204378

[B35] Ruben S. Garbe E. Mogavero S. Albrecht-Eckardt D. Hellwig D. Häder A.. (2020). Ahr1 and Tup1 Contribute to the Transcriptional Control of Virulence-Associated Genes in Candida Albicans. mBio 11. doi: 10.1128/mBio.00206-20 PMC7188989 32345638

[B36] Sancho S. Artero A. Zaragoza R. Camarena J. González R. Nogueira J. (2012). Impact of Nosocomial Polymicrobial Bloodstream Infections on the Outcome in Critically Ill Patients. Eur. J. Clin. Microbiol. Infect. Dis. 31, 1791–1796. doi: 10.1007/s10096-011-1503-8 22167257

[B37] Sheppard D. C. Yeaman M. R. Welch W. H. Phan Q. T. Fu Y. Ibrahim A. S.. (2004). Functional and Structural Diversity in the Als Protein Family of Candida Albicans. J. Biol. Chem. 279, 30480–30489. doi: 10.1074/jbc.M401929200 15128742

[B38] Sherry L. Rajendran R. Lappin D. F. Borghi E. Perdoni F. Falleni M.. (2014). Biofilms Formed by Candida Albicans Bloodstream Isolates Display Phenotypic and Transcriptional Heterogeneity That Are Associated With Resistance and Pathogenicity. BMC Microbiol. 14, 182–182. doi: 10.1186/1471-2180-14-182 24996549 PMC4105547

[B39] Silverman R. J. Nobbs A. H. Vickerman M. M. Barbour M. E. Jenkinson H. F. (2010). Interaction of Candida Albicans Cell Wall Als3 Protein With Streptococcus Gordonii SspB Adhesin Promotes Development of Mixed-Species Communities. Infect. Immun. 78, 4644–4652. doi: 10.1128/IAI.00685-10 20805332 PMC2976310

[B40] Singh S. Singh S. K. Chowdhury I. Singh R. (2017). Understanding the Mechanism of Bacterial Biofilms Resistance to Antimicrobial Agents. Open Microbiol. J. 11, 53–62. doi: 10.2174/1874285801711010053 28553416 PMC5427689

[B41] Todd O. A. Fidel P. L. Harro J. M. Hilliard J. J. Tkaczyk C. Sellman B. R.. (2019). Candida Albicans Augments Staphyloccus Aureus Virulence by Engaging the Staphylococcal Quorum Sensing System. mBio 10, e00910–e00919. doi: 10.1128/mBio.00910-19 31164467 PMC6550526

[B42] Tsui C. Kong E. F. Jabra-Rizk M. A. (2016). Pathogenesis of Candida Albicans Biofilm. Pathog. Dis. 74. doi: 10.1093/femspd/ftw018 PMC5975230 26960943

[B43] Valenza G. Tappe D. Turnwald D. Frosch M. König C. Hebestreit H.. (2008). Prevalence and Antimicrobial Susceptibility of Microorganisms Isolated From Sputa of Patients With Cystic Fibrosis. J. Cystic Fibrosis 7, 123–127. doi: 10.1016/j.jcf.2007.06.006 17693140

[B44] Van Dyck K. Viela F. Mathelié-Guinlet M. Demuyser L. Hauben E. Jabra-Rizk M. A.. (2021). Adhesion of Staphylococcus Aureus to Candida Albicans During Co-Infection Promotes Bacterial Dissemination Through the Host Immune Response. Front. Cell. Infect Microbiol. 10, 624839–624839. doi: 10.3389/fcimb.2020.624839 33604309 PMC7884861

[B45] Vila T. Kong E. F. Montelongo-Jauregui D. Van Dijck P. Shetty A. C. Mccracken C.. (2021). Therapeutic Implications of C. Albicans-S. Aureus Mixed Biofilm in a Murine Subcutaneous Catheter Model of Polymicrobial Infection. Virulence 12, 835–851. doi: 10.1080/21505594.2021.1894834 33682623 PMC7946022

[B46] Wang R. Braughton K. R. Kretschmer D. Bach T.-H. L. Queck S. Y. Li M.. (2007). Identification of Novel Cytolytic Peptides as Key Virulence Determinants for Community-Associated MRSA. Nat. Med. 13, 1510–1514. doi: 10.1038/nm1656 17994102

[B47] Young T. Alshanta O. A. Kean R. Bradshaw D. Pratten J. Williams C.. (2020). Candida Albicans as an Essential “Keystone” Component Within Polymicrobial Oral Biofilm Models? Microorganisms 9. doi: 10.3390/microorganisms9010059 PMC7823588 33379333

