# Supplementary material for: Investigating the Transcriptome of Candida albicans in a Dual-Species Staphylococcus aureus Biofilm Model
Source: Front Cell Infect Microbiol. 2021 Nov 23;11:791523. doi: 10.3389/fcimb.2021.791523 (PMC8650683; doi:10.3389/fcimb.2021.791523)
Supplement: Supplementary file 1 [file Presentation_1.pptx]

## Slide 1
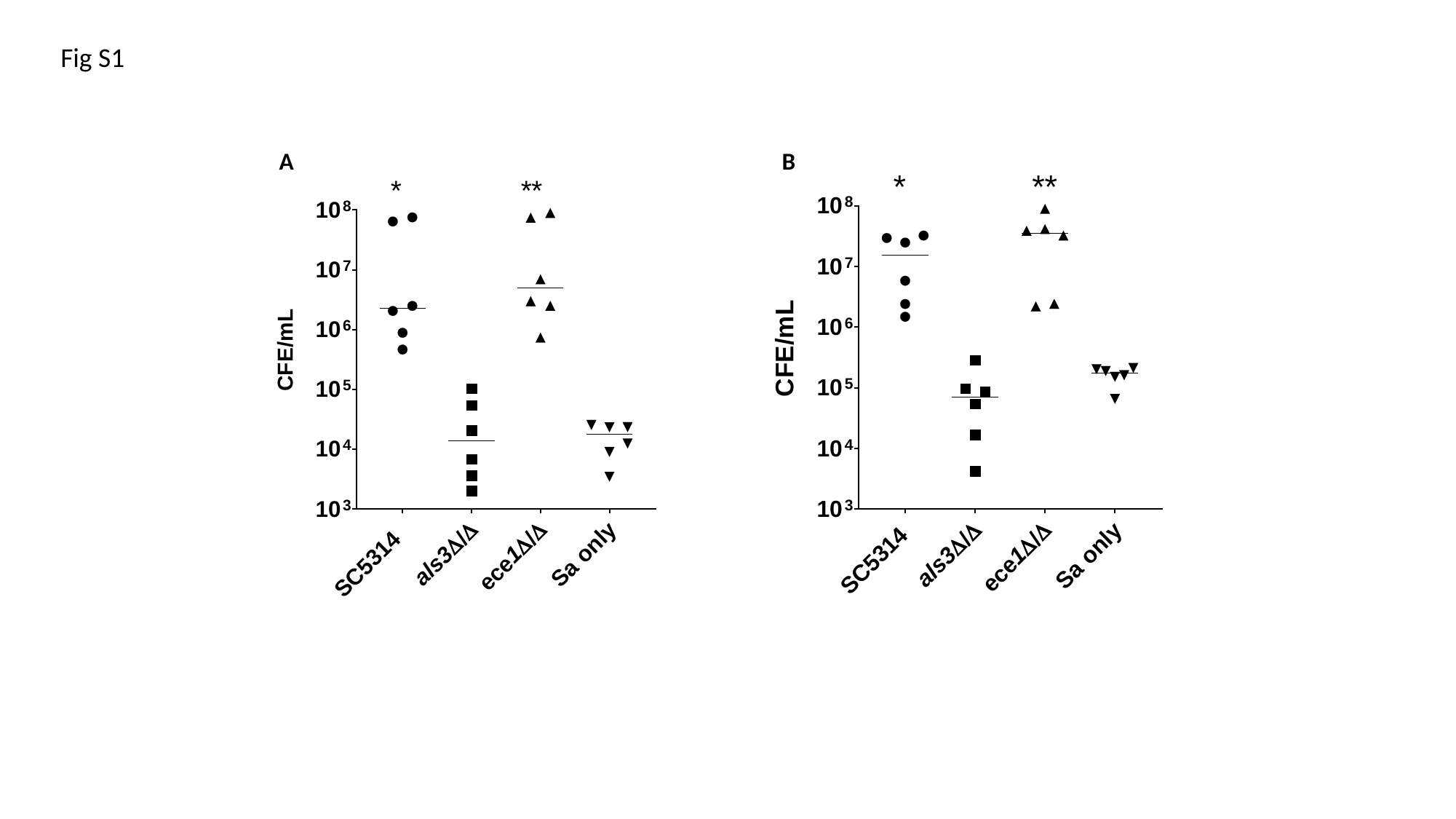

Fig S1
A
B

## Slide 2
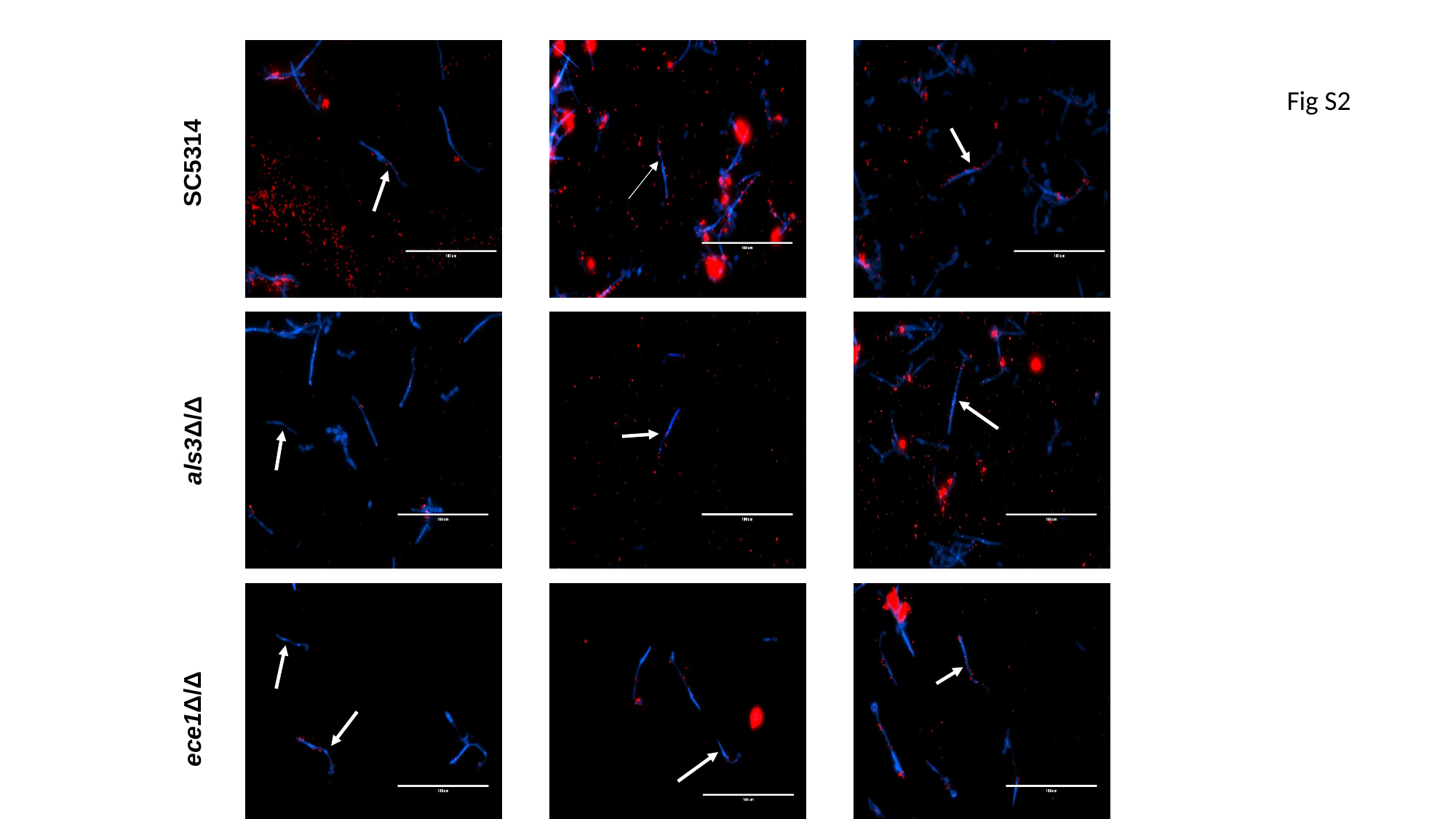

Fig S2
SC5314
als3Δ/Δ
ece1Δ/Δ

## Slide 3
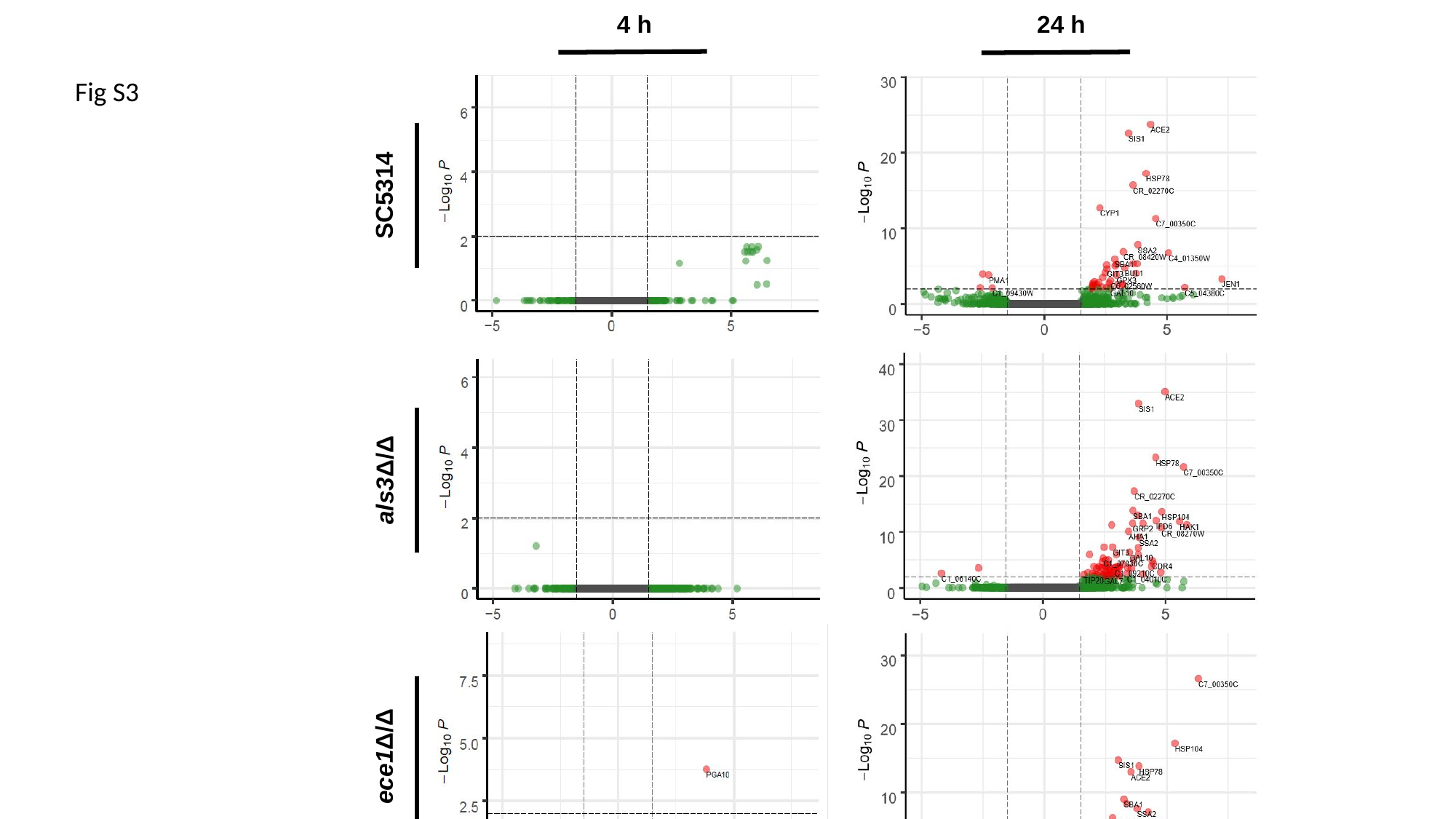

4 h
24 h
SC5314
als3Δ/Δ
ece1Δ/Δ
Fig S3

## Slide 4
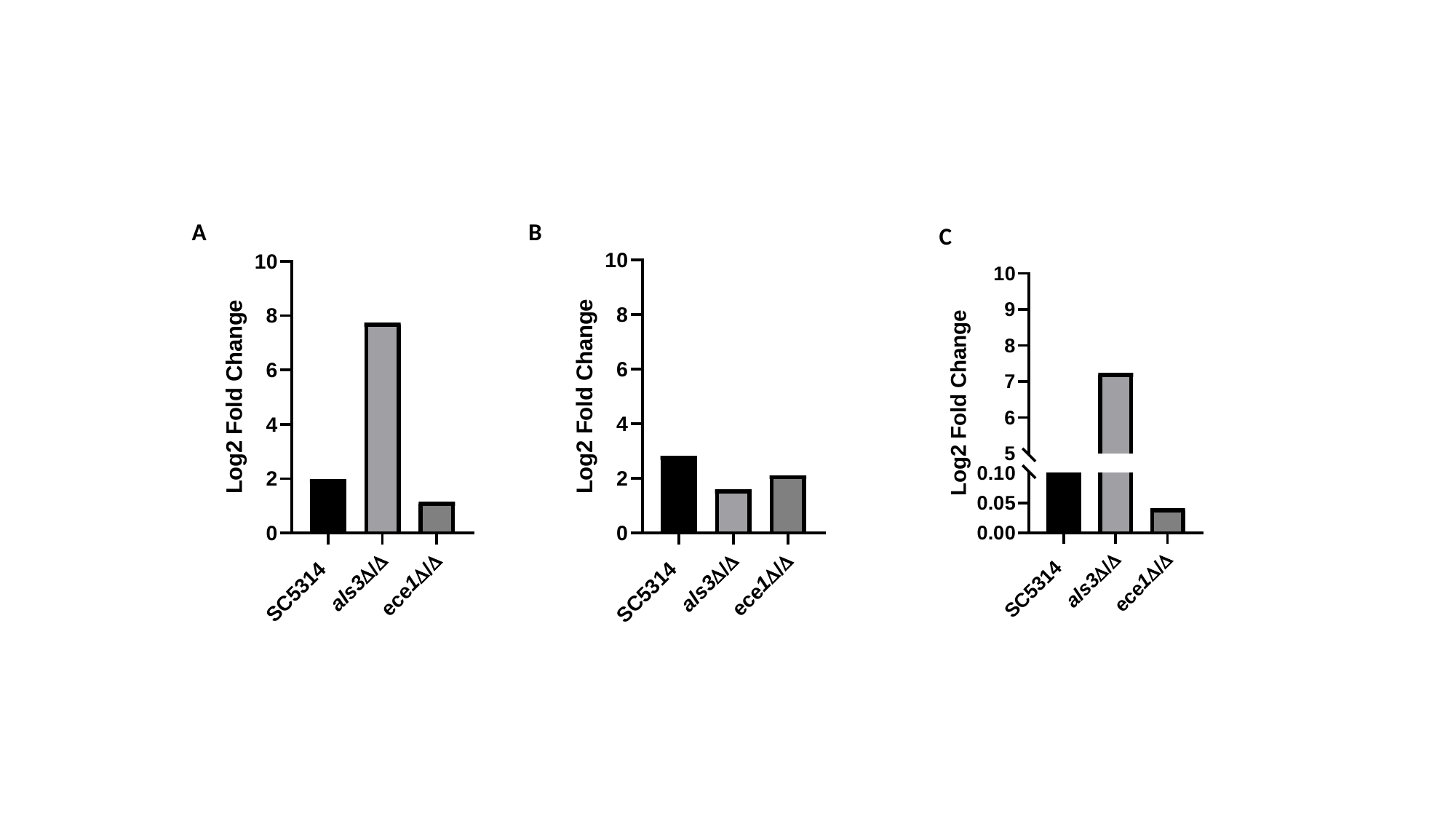

A
B
C

## Slide 5
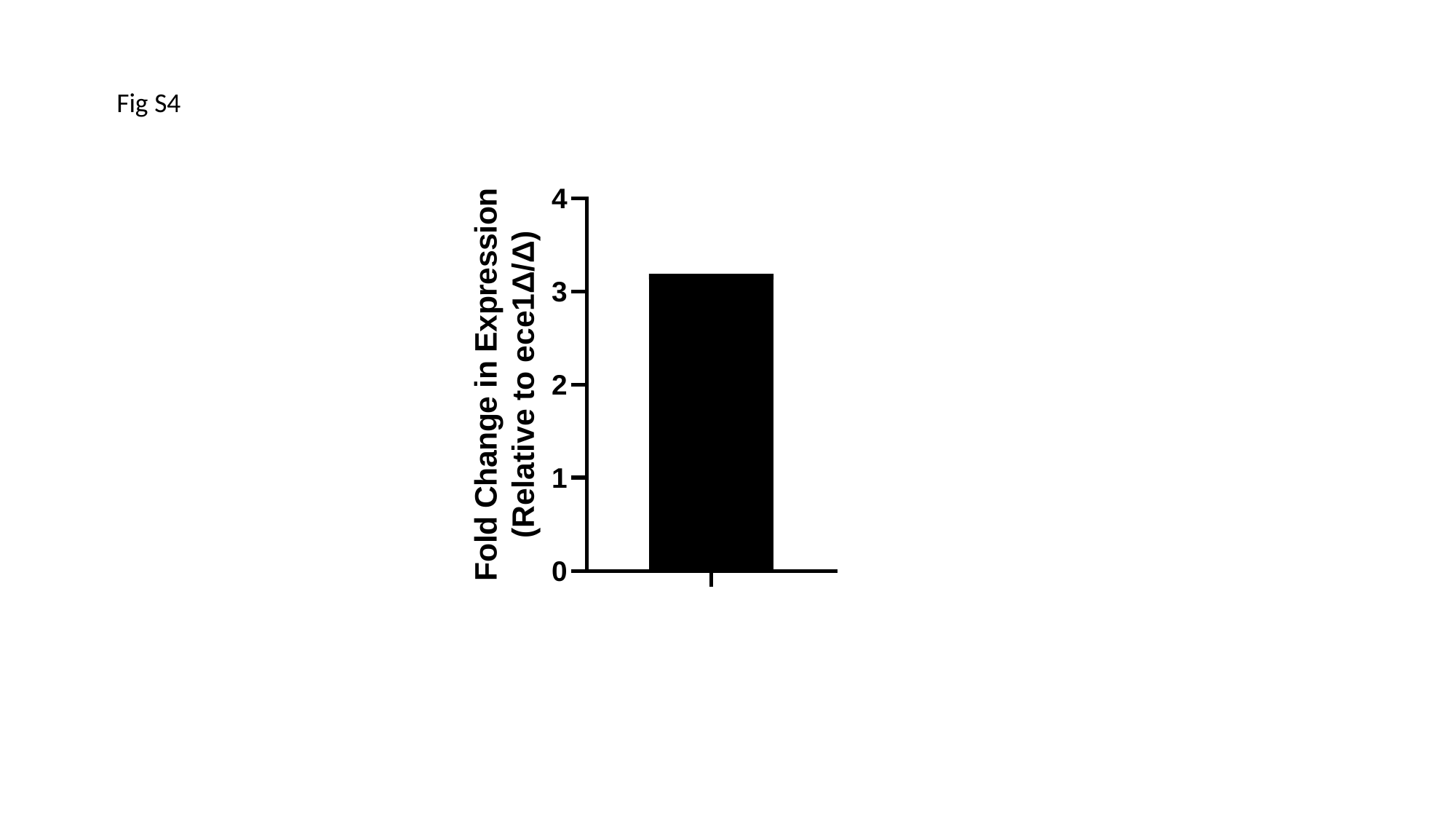

Fig S4

## Slide 6
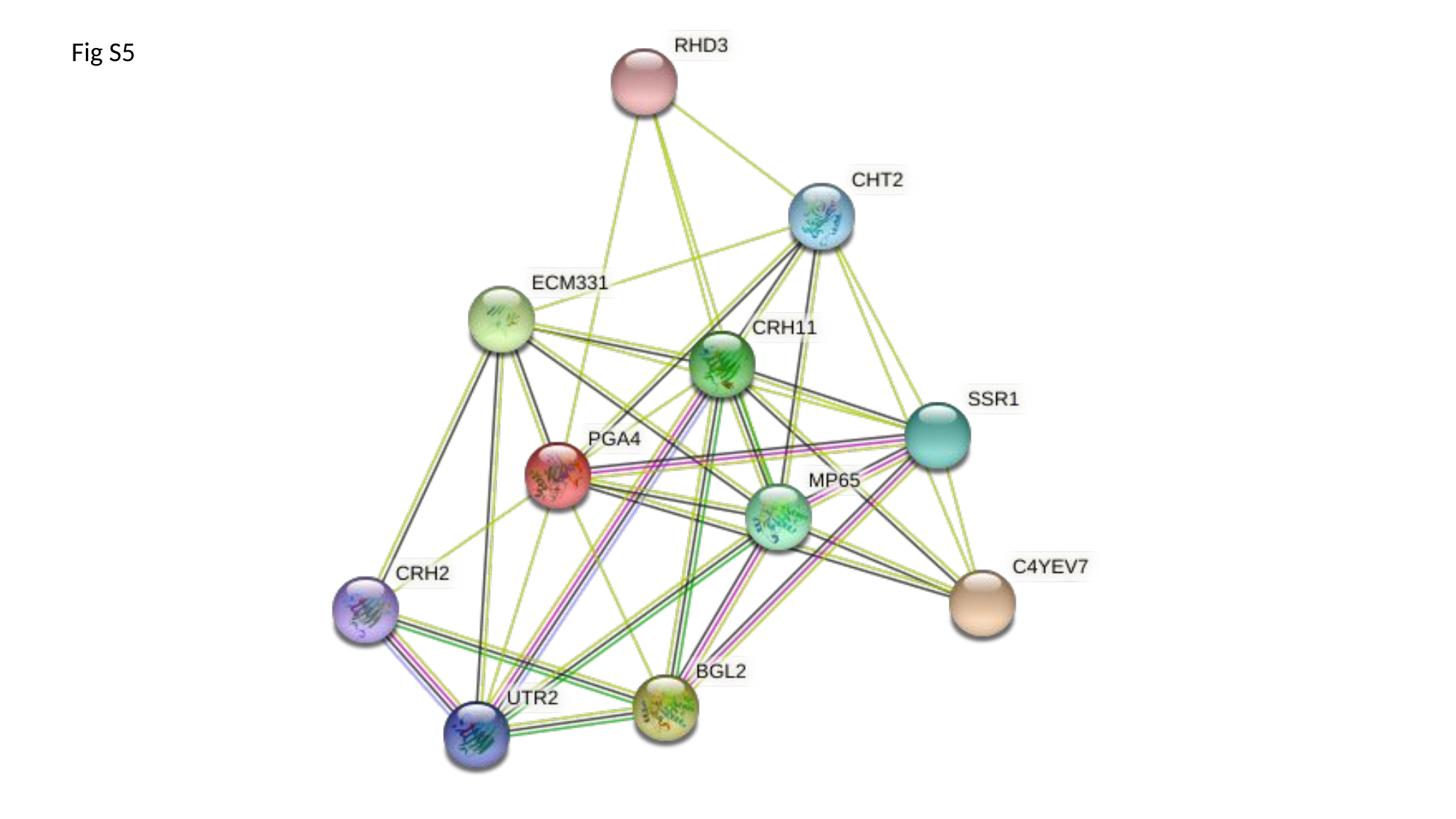

Fig S5
